# CLINICAL PROFILE OF CHILDREN WITH AND WITHOUT COMORBIDITIES HOSPITALIZED WITH COMMUNITY-ACQUIRED PNEUMONIA

**DOI:** 10.1590/1984-0462/2020/38/2018333

**Published:** 2020-05-08

**Authors:** Rafaela Baroni Aurilio, Clemax Couto Sant’Anna, Maria de Fátima Bazhuni Pombo March

**Affiliations:** a Universidade Federal do Rio de Janeiro, Rio de Janeiro, RJ, Brazil.

**Keywords:** Pneumonia, Child, Risk factors, Pneumonia, Criança, Fatores de risco

## Abstract

**Objective:**

To describe the clinical profile of children and adolescents hospitalized with community-acquired pneumonia (CAP). They were divided into two groups: those with and those without comorbidities.

**Methods:**

An observational, cross-sectional, descriptive study with prospective data collection, was carried out in a cohort of patients aged zero to 11, who were hospitalized with a clinical and radiological diagnosis of community-acquired pneumonia, from January 2010 to January 2012. As an exploratory study, the two groups were compared through logistic regression for possible risk factors associated with community-acquired pneumonia. Relative risk (RR) was used with a 95% confidence interval (95%CI). The process of selection for independent variables was stepwise forward, with a significance level of 5%.

**Results:**

There were 121 cases of community-acquired pneumonia evaluated, and 47.9% had comorbidities. In the bivariate analysis, patients with comorbidities demonstrated higher chances for: age >60 months (p=0.005), malnutrition (p=0.002), previous use of antibiotics (p=0.008) and previous hospitalization for community-acquired pneumonia in the last 24 months (p=0.004). In the multivariate analysis, these variables were independent predictors of community-acquired pneumonia in patients with the comorbidities: age >60 months (p=0.002; RR=5.39; 95%CI 1.89-15.40); malnutrition (p=0.008; RR=1.75; 95%CI 1.75-44.60); previous use of antibiotics (p=0.0013; RR=3.03; 95%CI 1.27-7.20); and previous hospitalization for community-acquired pneumonia (p=0.035; RR=2.91; 95%CI 1.08-7.90).

**Conclusions:**

Most patients with community-acquired pneumonia and comorbidities were aged >60 months, were malnourished, had used antibiotics and had been hospitalized for community-acquired pneumonia. Comorbidities were associated with a higher chance of malnutrition and hospitalizations for community-acquired pneumonia in an older age group, compared to children without comorbidities. Knowledge of this clinical profile may contribute to better assist pediatric patients with community-acquired pneumonia hospitalized in referral centers.

## RESUMO

**Objetivo:**

Descrever o perfil clínico de crianças e adolescentes hospitalizados com pneumonia adquirida na comunidade, separando-os em dois grupos: com e sem comorbidades.

**Métodos:**

Estudo observacional, transversal, descritivo, com coleta prospectiva de dados, realizado na coorte de pacientes de zero a 11 anos, hospitalizados com diagnósticos clínico e radiológico de pneumonia adquirida na comunidade de janeiro de 2010 a janeiro de 2012. De modo exploratório, buscou-se comparar os dois grupos por regressão logística quanto a possíveis fatores de risco para pneumonia adquirida na comunidade, por meio de risco relativo (RR) com intervalo de confiança de 95% (IC95%). O processo de seleção das variáveis independentes foi o de *stepwise forward*, a 5%.

**Resultados:**

Foram avaliados 121 casos; 47,9% tinham comorbidades. Na análise bivariada, pacientes com comorbidades mostraram maiores chances de apresentar: idade >60 meses (p=0,005), desnutrição (p=0,002), uso prévio de antibióticos (p=0,008) e hospitalização por pneumonia adquirida na comunidade nos últimos 24 meses (p=0,004). Na análise multivariada, estas foram variáveis preditoras independentes para pneumonia adquirida na comunidade, nos pacientes com comorbidades: idade >60 meses (p=0,002; RR=5,39; IC95% 1,89-15,40); desnutrição (p=0,008; RR=1,75; IC95% 1,75-44,60); uso prévio de antimicrobianos (p=0,0013; RR=3,03; IC95% 1,27-7,20) e hospitalização prévia por pneumonia adquirida na comunidade (p=0,035; RR=2,91; IC95% 1,08-7,90).

**Conclusões:**

A maioria dos pacientes com pneumonia adquirida na comunidade e comorbidades tinha idade >60 meses, desnutrição, fizera uso prévio de antibióticos e havia sido hospitalizado por pneumonia adquirida na comunidade. As comorbidades relacionam-se à maior chance de desnutrição e a hospitalizações por pneumonia adquirida na comunidade em faixa etária mais elevada do que crianças sem comorbidades. O conhecimento desse perfil clínico pode contribuir para melhor assistência da pneumonia adquirida na comunidade em pacientes pediátricos hospitalizados em serviços de referência.

## INTRODUCTION

Pneumonia is the leading infectious cause of death in children worldwide, accounting for the death of 920,136 children under five years old in 2015, which is 16% of all deaths in this age group.^1^ The worldwide estimate of the incidence of community-acquired pneumonia (CAP) among children under five in developing countries is about 0.22 episodes per child/year, with 11.5% of cases becoming severe. These data from 2010, compared to 2000, show a reduction in prevalence of around 25%, which is related to the decrease in risk factors for CAP in these countries.^1^ ^,^ ^2^ ^,^ ^3^ ^,^ ^4^ Rodrigues et al., in a retrospective study with data from the Computing Department of the Public Health System (*Departamento de Informática do Sistema Único de Saúde* - DATASUS), showed that, in Brazil as a whole, there was an average annual reduction in mortality rates per CAP from 1991 to 2007 in children under four years of age. In children under one year old or from one to four years old, the average annual reduction in mortality rates from pneumonia was 0.12 and 0.07, respectively.^5^

CAP may be defined by the presence of signs and symptoms, such as coughing, fever, tachypnea, dyspnea, chest pain or referred abdominal pain, wheezing from respiratory auscultation in a previously healthy child due to infection acquired outside the hospital. Wheezing-free tachypnea, with or without dyspnea, is the most common and important symptom for the diagnosis. In developing countries, the term “lower respiratory tract infection” may be adopted, as it does not require a radiological examination.^6^

The risk factors for host-related CAP, according to the literature, are: malnutrition, young age, comorbidities (such as congenital heart and lung disease), low birth weight, previous episodes of wheezing and pneumonia, lack of breastfeeding, incomplete series of vaccinations and viral respiratory infections.^7^ ^,^ ^8^

Most articles that involve risk factors for childhood CAP exclude patients with comorbidities from their analysis, thus making it difficult to compare groups with CAP that also may or may not have an underlying disease. This study aimed to describe the risk variables for CAP in hospitalized children with CAP, separating them into two groups: those with comorbidities and those without.

## METHOD

A cross-sectional study with prospective data collection was conducted with children aged zero to 11 years old, who were hospitalized with clinical and radiological diagnoses of CAP, from January 2010 to January 2012. The study was carried out in the wards of the Pediatrics Department of the Martagão Gesteira Institute of Childcare and Pediatrics (*Instituto de Puericultura e Pediatria Martagão Gesteira* - IPPMG), a unit of the *Universidade Federal do Rio de Janeiro* (UFRJ), which provides outpatient and hospital care for children ages 0 to 11 years old. IPPMG has outpatient clinics from all pediatric specialties, thus generating a greater number of customer care opportunities and hospitalizations of patients with chronic diseases in their beds. There are six wards, one for patients with onco-hematological diseases and the other for pre- or postoperative patients with general pediatric surgical diseases. The others were hospitalized for various diseases. It is worth noting that, because they are at a university level, they have a lot of comorbidities, with the most prevalent underlying diseases being: encephalopathies, hemoglobinopathies and onco-hematological pathologies. There are eight beds in each ward, except for the hematology ward, where there are six beds, making a total of 46 beds. More details about the study site were described by Ferreira et al.^9^

All patients hospitalized with CAP during the referred period were studied. Data were collected with the caregiver, who completed the questionnaire at the time of the interview. The length of hospitalization of each case was identified by reviewing the medical records after the patient was discharged from the hospital. The definition of CAP adopted in this study was acute pneumonia acquired outside the hospital environment. Inclusion criteria were: children aged zero to 11 years old, hospitalized with a clinical presentation of cough, fever, tachypnea, dyspnea, chest or abdominal pain for less than seven days and a chest radiography showing an image compatible with pneumonia (segmental or lobar alveolar patterns, an air bronchogram, abscesses, pneumatoceles, pleural effusion, interstitial infiltrates and atelectasis).^3^ Patients hospitalized in the intensive care unit (ICU), in the emergency room, and those transferred to another institution while previously included in this study, were excluded. Cases of chronic pneumonia (pneumonia lasting more than three weeks), refusal to sign the informed consent form and the consent form for the children and adolescents, and lack of agreement between the pediatrician and the radiologist in the radiological diagnosis of the chest were also excluded. The same radiologist evaluated all of the patients’ radiographs.

An adapted form of the Risk Factors Investigation Fact Sheet of the Caribbean prospective multi-center study was developed simultaneously in 12 health centers in three countries (five centers in Brazil, six in Argentina and one in the Dominican Republic), from July 1998 to December 2002. It aimed to evaluate the *in vitro* and *in vivo* resistances of Streptococcus pneumoniae in PAC.^10^ In Brazil, the IPPMG was one of the centers chosen for the study, and data continue to be collected in their wards today.

The patients were divided into groups with and without comorbidities (Groups 1 and 2, respectively). The following host-related variables were evaluated:

- Clinical - nutritional status, classifying the patient as eutrophic, malnourished (below the third percentile on the National Center for Health Statistics (NCHS) curve) or in the risk zone (between the third and tenth percentile on the NCHS curve); previous antibiotic use before hospitalization (up to 90 days prior to hospitalization) and reason for use; previous hospitalization for CAP in the last 24 months; previous wheezing in the last 12 months; and exclusive breastfeeding time (period of only breastfeeding, with no use of formulas or any other type of food; evaluated in children from six to 24 months old).
- Demographic - sex, patient’s age and number of patients older than 60 months.

A database was developed in the Microsoft Office Excel program and was then analyzed with the Statistical Analysis System (SAS) software, version 6.11. Then, a descriptive analysis was performed for numerical data expressed as an average ± standard deviation (SD) and median; frequency (n) and percentage (%) for categorical data. For the exploratory bivariate analysis, the prevalence ratio (PR) between the exposure or lack of exposure to the variables in both groups was calculated, with a 95% confidence interval (95%CI). Logistic regression analysis was performed to evaluate the simultaneous influence of host-related variables in children and adolescents regarding the occurrence of CAP in cases with comorbidities. The independent variables selection process used was stepwise forward, at a significance level of 5%. The project was approved by the Research Ethics Committee of the IPPMG/UFRJ, under number 42/09.

## RESULTS

One hundred and twenty-four patients were studied and three were excluded due to incomplete data from the medical records. Therefore, a total of 121 patients were analyzed. In the descriptive analysis of the studied population, 63 (52.1%) had no comorbidities (Group 2) and 58 (47.9%) had comorbidities (Group 1), the most frequent being hemoglobinopathies (11.6% -- 14 cases) and encephalopathies (10.7% -- 13 cases). The remaining 31 cases (25.6%) were: wheezing infants and asthma (7/58), AIDS (5/58), congenital airway malformation (3/58), prematurity (3/58), congenital immunodeficiencies (2/58) and others (11/58). There were 69 (57%) male patients. The average age of the population was 40.6±35.8 months (median = 32 months), and the majority of the population was under five years old. The mean age was 53±40 and 29±27 months in Groups 1 and 2, respectively (p<0.05). With regard to hospitalization length, the average stay was 12.1±19.6 and 8.2 ± 3.7 days, in Groups 1 and 2, respectively, but there was no statistically significant difference (p*=*0.19) between both groups.

Associations between host-related variables in Groups 1 and 2 are described in Table 1. Table 2 provides the parameters of the significant variables selected by the logistic regression method for PAC in Group 1.

**Table 1:** Associations between clinical and demographic variables related to community-acquired pneumonia with groups that did and did not have comorbidities (Groups 1 and 2).

Variable	Category	Group 1		Group 2		p-value^a^	PR (95%CI^b^)
		n	%	n	%		
Age ≥60 months	≥60 months	20	34.5	8	12.7	0.005	3.62 (1.46-9.06)
Nutritional status	Eutrophic	36	62.1	53	84.1	0.002	1
	Malnourished	14	24.1	2	3.2	0.002	10.3 (2.21-48.1)
	Risk Zone	8	13.8	8	12.7	0.002	1.47 (0.51-4.28)
Sex	Male	35	60.3	34	54	0.47	1.3 (0.63-2.67)
Previous use of antibiotics	Yes	37	63.8	25	39.7	0.008	2.67 (1.20-5.97)
Hospitalization for pneumonia in the last 24 months (1 without information)	Yes	21	36.8	9	14.3	0.004	3.50 (1.44-8.50)
Wheezing in the past 12 months	Yes	31	53.5	23	36.5	0.061	1.99 (0.96-4.13)
	No	27	46.6	40	63.5		
Exclusive breastfeeding (Children 6 to 24 months)	<4 months	5	35.7	9	39.1	0.83	0.86 (0.22-3.43)
	≥4 months	9	64.3	14	60.9		

n: number of cases; ^a^Fisher’s chi-square or exact test; ^b^95% confidence interval for the prevalence ratio (PR).

**Table 2:** Association of risk variables for community-acquired pneumonia with comorbidities.

Predictor Variable*	Coefficient	CSE	p-value	PR	95%CI
Age ≥60 months	1.68	0.53	0.002	5.39	1.89-15.40
Malnourished	2.17	0.82	0.008	8.83	1.75-44.60
Risk Zone	0.32	0.60	0.59	1.39	0.42-4.60
Previous use of antibiotics	1.10	0.44	0.01	3.03	1.27-7.20
Hospitalization for pneumonia in the last 24 months	1,06	0.50	0.03	2.91	1.08-7.90

*significant variables selected by the logistic regression method for CAP in the group with comorbidities; CSE: coefficient standard error; PR and 95% CI: prevalence ratio and its respective 95% confidence interval.

In the bivariate analysis, no statistically significant differences were observed between the groups regarding wheezing in the last 12 months (p = 0.06) and exclusive breastfeeding time (p = 0.83). There were statistically significant differences between the groups regarding age older than 60 months (p = 0.005), malnutrition (p = 0.002), prior antibiotic use (p = 0.008), prior hospitalization for pneumonia (p = 0.004) and completed basic vaccination (p = 0.021).

Logistic regression analysis was performed with the variables shown in Table 2. Multivariate analysis showed that age older than 60 months, malnutrition, previous antimicrobial use and previous hospitalization for CAP were independent predictors for CAP in Group 1.

## DISCUSSION

The present study describes clinical aspects of patients with and without comorbid CAP, who were hospitalized in the wards of a university pediatric hospital. A relevant frequency of the following risk factors was demonstrated in the general population: previous antimicrobial use, mainly by the CAP; lack of exclusive breastfeeding for at least four months; under 60 months and male.

Approximately half of the sample had comorbidities. In a study conducted in Tanzania involving 100 hospitalized children with CAP, 32% of the patients had comorbidities, with malaria, typhoid fever and anemia being the most prevalent. This was related to the greater severity and complications of CAP. This study also did not find longer hospital stays in patients with CAP and comorbidities.^13^ Another study conducted in 14 hospital units in Kenya intentionally selected two main groups with high and low prevalence of malaria, and assessed mortality in children under five, hospitalized for non-severe CAP (severity rating based on criteria from the World Health Organization (WHO), and associated with clinical criteria and penicillin monotherapy). The authors describe that 31.4% of patients had comorbidities upon being admitted, such as malaria, dehydration, diarrhea and significant anemia. Comorbidities were predictive factors for mortality.^14^ Regarding the most severe CAP, a study conducted in a tertiary hospital in South Africa involving 237 one-month-old children aged 18 years old, hospitalized in the ICU with CAP, showed that the presence of comorbidities was associated with worse clinical outcomes, with regard to mechanical ventilation time and ICU stay.^15^

In the assessment of the possible association of risk variables analyzed with Group 1, the most prevalent were: malnutrition, previous antimicrobial use and previous hospitalization for CAP. The group was 3.6 times more likely than Group 2 to be > 60 months old (this age range was an independent variable for CAP in this group), with PR = 5.39. This is probably due to the fact that, with the evolution of underlying diseases, new infections and hospitalizations are favored over time.

Although most children were eutrophic, there was a greater chance of malnutrition in Group 1. This was an independent variable in this group, probably due to cases of chronic diseases that are concomitant with undernutrition. Malnutrition has been reported as a risk variable for CAP acquisition, hospitalization and mortality. Teepe et al. demonstrated that low weight for age and low weight for height were related to the increased risk of CAP.^16^ In Brazil, a study showed that the lower the weight/age nutritional score, the greater the chance of hospitalization for CAP.^17^ Another study, however, found no association between nutritional status and CAP complications.^18^ In the meta-analysis of Chisti et al., the Odds Ratio for CAP mortality in severely malnourished children ranged from 2.5 to 15.1, compared to undernourished children.^19^ Similarly, in India, severe malnutrition was also related to death from CAP.^20^ A cited study from East Africa (Kenya) showed that acute malnutrition (Z-score <-3) and young age (under 11 months) were among the top five mortality-related variables in children under five years old hospitalized for non-severe CAP.^14^

After a comprehensive literature review, few studies describing risk variables related to CAP in children with underlying diseases were found. The meta-analysis performed by Jackson et al. in children hospitalized for risk factors related to lower respiratory tract infections (CAP or bronchiolitis), showed a significant association of respiratory disease with: low birth weight, air pollution, malnutrition, incomplete immunization in the first year, HIV, lack of breastfeeding and crowding at home.^21^

In the present study, just over half of the cases had previously used antimicrobials, mainly due to previous CAP. The bivariate analysis showed that the risk of previous antibiotic use was 2.67 times higher in Group 1 patients than in Group 2 (95%CI 1.20-5.97), probably because they experienced a higher occurrence of respiratory infections requiring antimicrobial therapy. From the multivariate analysis, prior antibiotic use was an independent variable for PAC in Group 1 patients, as well as previous hospitalization for PAC, with a PR of 3.03 and 2.91, respectively. A similar finding was found in a case-control study in New Zealand, where previous respiratory infections in children or families led to a higher risk of acquiring CAP.^22^ In Campinas, children hospitalized with CAP (with and without complications) had a 5.49 higher risk of complications due to previous and indiscriminate use of antimicrobials.^23^

Hospitalization for PAC in the last 24 months was more associated with comorbidities in Group 1 and was an independent variable for CAP in this group, with a PR of 2.91. Recently, the importance of comorbidities influencing the severity of CAP was related to congenital heart disease in a pediatric ICU study in China.^24^ Our data showed that the most prevalent comorbidities were encephalopathies and hemoglobinopathies. Of these, sickle cell disease was the most common, as there is a high tendency for early infections from *S. pneumoniae* and *H. influenzae* up to five years of age. Sickle cell disease progresses with auto-splenectomy, due to the presence of thrombi and infarctions, which culminate in atrophy and fibrosis of the spleen, predisposing individuals to infection from encapsulated germs.^25^ In the group of encephalopathies, non-progressive chronic encephalopathy (NPCE) was the most frequent, due to its potential risk of recurrent CAP, because, generally, PNEC accompanies bronchial obstruction, predisposing individuals to lower respiratory tract infections. Such patients may have even greater CAP severity, prolonged hospitalization and longer antibiotic therapy.^26^

Wheezing in the past 12 months was present in almost half of the group with comorbidities, but there was no statistical association. The presence of a previous episode of wheezing may correspond to an underlying disease, such as asthma, or episodes of viral respiratory infections. Some authors have shown that infants with one or more wheezing episodes in the first three months of life and children and adolescents with asthma had a higher risk of developing CAP, although this was not an objective of the present study.^16^ ^,^ ^27^ In southern Brazil, a case-control study in Pelotas showed that previous wheezing was the second most important risk factor for hospitalization for respiratory disease.^28^

Although breastfeeding has a protective effect against childhood respiratory diseases, especially in the early years, as it is related to the amount of breast milk received and the time of breastfeeding, most children in our study, under 24 months of age, were exclusively breast-fed for less than four months. However, this data was not relevant in either group. A limitation to this variable was the fact that the person interviewed was not always the mother, which may have hindered the reliability of the information provided. In the US, exclusive breastfeeding for a period of six months or more promoted greater protection against respiratory tract infection.^29^ In Brazil, a study of children under one year of age showed that breastfeeding led to a reduction in hospitalization rates for CAP.^30^

The present study had some limitations. Due to its cross-sectional design, it was not possible to further accompany the patients in order to identify CAP sequelae and eventual readmissions. In addition, to assess the associations of variables in the groups with and without comorbidities, all underlying diseases of different severity levels were included, due to the small number of cases in each comorbidity subgroup. Other limitations were possible respondent memory bias at the time of the patients’ hospitalization, as well as the reduced number of patients during the months of collection. This was because the wards were temporary closed for two months due to administrative problems. On the other hand, the present study was developed in a tertiary hospital and included all CAP patients hospitalized in the study period, with a single investigator, and standardized care at the institution. It is also worth considering that our data were collected about seven years ago and that the manner of giving assistance during this period did not undergo major changes in the institution. Thus, it is worth acknowledging that the clinical knowledge of children and adolescents with CAP and comorbidities contained in this study may contribute to better assistance of pediatric patients hospitalized in referral services.
